# Supplementary material for: Content-rich biological network constructed by mining PubMed abstracts
Source: BMC Bioinformatics. 2004 Oct 8;5:147. doi: 10.1186/1471-2105-5-147 (PMC528731; doi:10.1186/1471-2105-5-147)
Supplement: Additional File 2 — The original results of the above study (non-essential files are deleted to keep the file size under the limit set by BMC bioinformatics). [file 1471-2105-5-147-S2.bz2 › chilibotAdditionalFile2/dip05/318ID10583954E2439/html/GSN_ACTB.html]

 


 **GSN** and **ACTB** 
  
Found 10 abstracts in PubMed, retrieved 05.  
 

 What does Google say? 
 PDF only 
| .edu only 

---

**Interactive relationship** (e.g. stimulation, inhibition, etc)

**Non-interactive relationship** (e.g. studied together, co-existance, homology, etc.)

- Phosphorylation of gelsolin  [ **GSN** ]  in the gelsolin  [ **GSN** ]  actin2  [ **ACTB** ]  complex was inhibited by 90%.  Ref: 10210201 Protein Sci, 1999
- beta Actin  [ **ACTB** ]  and gelsolin  [ **GSN** ]  associated transiently with beads followed by LAMP 2 and cathepsin B.  Ref: 10945978 J Biol Chem, 2000
